# Identification of Hub Genes in Protective Effect of Astragaloside IV on Aconitine-Induced Cardiac Damage in Zebrafish Based on Bioinformatics Analysis

**DOI:** 10.3389/fphar.2020.00957

**Published:** 2020-06-24

**Authors:** Mingzhu Wang, Yanan Shi, Lei Yao, Qiang Li, Youhua Wang, Jianhua Li, Deyu Fu

**Affiliations:** ^1^ Yueyang Hospital of Integrated Traditional Chinese and Western Medicine, Shanghai University of Traditional Chinese Medicine, Shanghai, China; ^2^ Children’s Hospital of Fudan University, Fudan University, Shanghai, China; ^3^ Longhua Hospital, Shanghai University of Traditional Chinese Medicine, Shanghai, China

**Keywords:** astragaloside IV, aconitine, zebrafish, cardiac damage, bioinformatics analysis, RNA sequencing

## Abstract

Accumulating evidence suggests that Astragaloside IV (AS-IV) improves cardiac function and protects the cardiovascular system. However, the molecular targets involved remain ambiguous. In this work, we report research suggesting that AS-IV can antagonize arrhythmias and reduce the cardiac damage induced by aconitine in zebrafish. Zebrafish have certain benefits with respect to studying the effect of drugs on cardiovascular disease. The possible mechanisms involved are analyzed, and hub gene targets are predicted. First, a model of cardiac damage induced by aconitine was created, and then a safe drug concentration of AS-IV was screened, and the appropriate drug dose gradient was selected within a safe drug concentration range. Second, we confirmed the protective effect of AS-IV in the cardiovascular system by observing changes in zebrafish heart rates and the cardiac and vascular structure. Third, we aimed to demonstrate the antagonistic mechanism of AS-IV on heart rate and cardiac damage induced by aconitine in zebrafish, with differentially expressed genes (DEGs) detected by RNA sequencing. The DEGs were then further analyzed by bioinformatic techniques, such as function enrichment analysis, protein-protein interaction network, and DNA-microRNA networks, for example. Next, we predicted the hub genes of the cardiac protective effects of AS-IV. Finally, we validated these genes in different transcriptome sequence datasets of cardiac damage. Thus, we conclude that miR-26b-5p/ATF3/JUN are key targets of AS-IV and play an important role in maintaining cardiac homeostasis and regulating cardiac remodeling.

## Introduction

Cardiovascular disease (CVD) may be considered a series of pathological conditions involving the heart and circulatory system at all levels. In recent years, CVD has become a major threat because of its high morbidity, disability, and mortality (Benjamin et al., 2018). However, the molecular mechanisms of CVD have not been clearly elucidated. A better understanding of the factors that result in the occurrence and development of CVD could reveal potential therapeutic targets for the disease.

Radix Astragali (*Astragalus mongholicus* Bunge) is an important herb that has been used in the clinic in China for more than 2,000 years. Radix Astragali is known as Huangqi in Traditional Chinese Medicine, and its protective effect on the cardiovascular system (including a cardiotonic effect, cardiomyocyte protection, and blood pressure regulation), has become the focus of fundamental and clinical research (Ren et al., 2013; Li et al., 2017a; Zang et al., 2020). Astragaloside IV (AS-IV), a key bioactive component of Radix Astragali, has attracted increasing attention over recent years due to its potential therapeutic benefits in terms of improving cardiac function and protecting the myocardium (Zhao et al., 2012). The study of its pharmacological effects and mechanism of cardiovascular protection will contribute to the further development and utilization of AS-IV.

The latest reports show that AS-IV could alleviate heart failure by promoting angiogenesis through the Janus kinase/signal transducers and activators of transcription (JAK/STAT) 3 pathway (Sui et al., 2019), as well as alleviating doxorubicin-induced cardiomyopathy by inhibiting nicotinamide adenine dinucleotide phosphate (NADPH) oxidase-derived oxidative stress (Lin et al., 2019), and protecting cardiac function after myocardial infarction by regulating the phosphatase and tensin homolog/phosphoinositide 3-kinase/protein kinase B (PTEN/PI3K/Akt) signaling pathway (Cheng et al., 2019). These findings indicate that further in-depth studies on the mechanism of AS-IV in the treatment of heart disease are of great significance and provide the groundwork for clinical interventions at a later stage. In contrast to the above experiments, we used zebrafish as a model and utilized high-throughput sequencing and bioinformatic technology to screen and verify the expression of hub genes.

The zebrafish is one of the most important model vertebrates currently used in the field of drug screening and toxicity assessment in CVD (Asnani and Peterson, 2014), for several reasons. First, zebrafish embryos and young fish have transparent bodies, meaning that their hearts can be clearly imaged under the microscope, while their heart rates and blood circulation are also clearly visible and easy to observe. Second, zebrafish share similar physiological and biochemical characteristics with mammals, with a genome that is up to 87% similar to the human genome, and can therefore be used to study the molecular mechanism of human diseases or in high-throughput drug screening (Howe et al., 2013). Third, cardiac fluorescence transgenic (cmlc2:GFP) zebrafish emit green fluorescence under a fluorescence microscope, which improves the accuracy and convenience with which changes in zebrafish heart morphology and structure may be observed (Gut et al., 2017). When studying drug interventions for CVD, changes in the structure of zebrafish hearts can be observed at different time periods.

As transcriptome sequencing technology has developed and matured, high−throughput platforms have become widely applied in gene expression analysis. In particular, RNA sequencing (RNA-seq) has been widely used in the diagnosis and treatment of CVD (Conesa et al., 2016). Screening the hub genes from the large amount of genetic data generated by RNA-seq relies on bioinformatic technology, which solves biological problems using several methods including applied mathematics, informatics, statistics, and computer science (Li et al., 2018). Given the importance of microRNA (miRNA) as a regulatory molecule in gene regulatory networks, further confirmation and functional analysis of the miRNAs involved in cardiac homeostasis will provide more effective therapeutic targets for the treatment of CVD.

**Figure 1:**
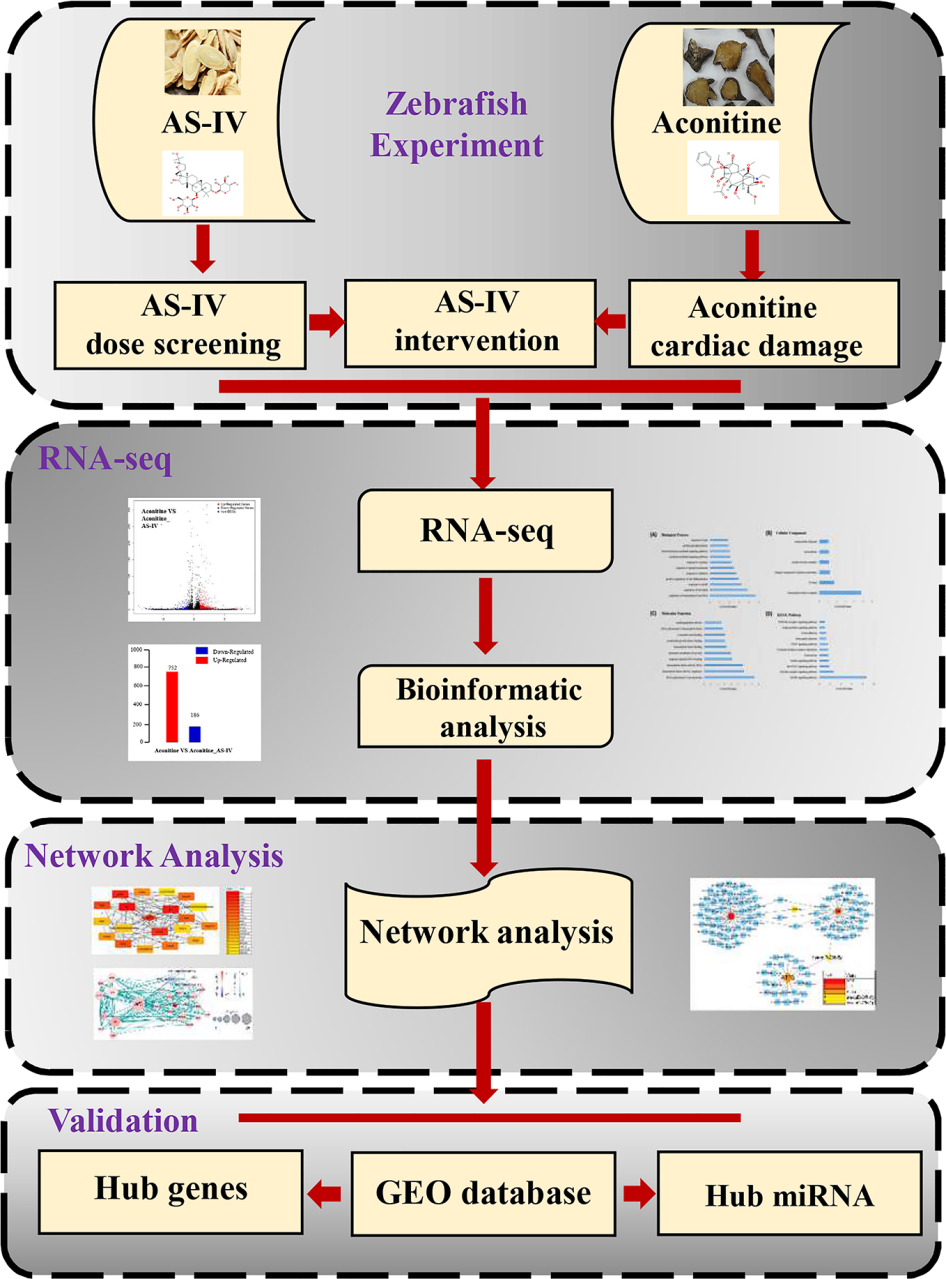
Workflow for AS-IV intervention in aconitine-induced cardiac damage in zebrafish.

The aim of present study was to verify whether AS-IV could protect against the cardiac damage induced by aconitine, and to explore the molecular mechanisms of its cardiac protective effect. First, we discuss the antagonistic effects of AS-IV at different concentrations on aconitine-induced cardiac damage in zebrafish by observing changes in heart rate, cardiac morphology, and the distance between the sinus venous and bulbus arteriosus (SV-BA). Second, we discuss the use of high−throughput sequencing technology to detect the differentially expressed genes (DEGs) of AS-IV in heart protection, followed by data mining, processing, and bioinformatics analysis to determine potential signaling pathways and gene targets. Finally, we validate these genes in different transcriptome sequence datasets of cardiac damage in the Gene Expression Omnibus (GEO) database. Thus, we present the hub genes associated with AS-IV intervention in aconitine-induced cardiac damage in zebrafish. The specific workflow followed is represented in **Figure 1**.

## Materials and Methods

### Zebrafish

Zebrafish, transgenic (cmlc2: GFP) Tübingen (TU) strain, belonging to the Danio family of Cyprinidae, were provided by the Institute of Pediatrics, Children's Hospital of Fudan University. The heart of a young zebrafish glows green under a fluorescence microscope, which facilitates the observation of zebrafish hearts and the measurement of related indicators. Adult zebrafish were bred in a water temperature of 28°C, at a pH of 7.0 to 7.5, and conductivity of 400–600 μs/cm. Zebrafish were fed three times a day, and exposed to fluorescent light for 14 h and to darkness for 10 h (Westerfield, 1994). Embryos were carefully collected at 10-min intervals to ensure precise developmental timing within a group. Embryos were submerged in culture medium with cautious and placed in an incubator at 28°C on a 14 h light, 10 h dark cycle. All procedures were approved by the Children's Hospital of Fudan University.

### Drug Configuration

AS-IV (99.24%) and aconitine (98.01%) were purchased from Chengdu Must Bio-Technology Limited, preserved at 4°C in a sealed environment, and protected from light. Dimethyl sulfoxide (DMSO) was used to facilitate dissolution. Ten milligrams AS-IV and 10 mg aconitine were mixed into 100 μl DMSO to form the mother liquor, which was diluted before use according to the required concentration of zebrafish culture fluid.

### Groups and Experimental Scheme

#### Model of Cardiac Damage Induced by Aconitine

At 48 hpf, the embryos were divided into a control group and an aconitine group, with 20 embryos in each group. The embryos were then placed into six wells, with an aconitine concentration of 15mg/L (Fang et al., 2012). At 72 hpf, the embryos were returned into ordinary culture medium and placed in an incubator at 28°C in a six-well plate, with each well containing 4 mL of liquid. Next, relevant indicators, including heart rate and SV-BA distance, were observed and recorded. The experiment was repeated twice.

#### Safe Concentration Screening of AS-IV

The collected embryos (6 hpf) placed in a 12-well plate, and each well contains 20 embryos and 4 ml culture medium with a different drug concentration. They were then placed in incubators, and the culture medium was changed every 24 h, three times in total. At 24 hpf, 48 hpf, 72 hpf, and 96 hpf, the number of zebrafish deaths, changes in heart morphology, and blood cell accumulation were observed and counted under a microscope.

#### Intervention of AS-IV

The 48 hpf-embryos were divided into five groups, with 20 embryos in each group, as follows: control group, aconitine group, and AS-IV 10/25/40 mg/L groups. In the aconitine group, embryos were placed in 15 mg/L aconitine medium. In the three AS-IV groups, 10, 25, and 40 mg/L AS-IV solution, respectively, was added into 15 mg/L aconitine medium. The remaining procedures were as described above.

### Zebrafish Development Observation and Recording

All embryos were reared at 28°C and heart rates (beats per minute, bpm) were measured at room temperature **(Supplementary Material, Video 1)**. Prior to the measurements, each dish was removed from the incubator and placed under the microscope light for 4 min at room temperature, allowing the embryos to acclimatize to the light and eliminate the effect of any startle response. The embryos were then anesthetized with tricaine (Wilson et al., 2009).

The embryos were subsequently adjusted to a side-lying position with cautious use of a needle. Ten embryos were collected in each treatment group. The heart beats of the embryos were recorded by counting beats per minute on the live video, as previously described (Keßler et al., 2015). The blood enters the atrium *via* the SV, and leaves the ventricle *via* the BA. During normal development, the atrium and ventricle overlap from the side view. However, if the development process is blocked, the position of the atrium and ventricle changes, and the distance between the SV and BA also changes accordingly. Therefore, the SV-BA distance can quantitatively reflect the degree of influence of drugs on the heart of zebrafish (Du et al., 2015). SV-BA distance was measured by Image J (http://www.rsb.info.nih.gov/ij/), as previously described.

### Data Analysis

All data were plotted using GraphPad Prism 7.0. Data were expressed as mean ± standard error of the mean (SEM), and the statistical software SPSS24.0 was used for statistical analysis. The t-test was used for comparison between the two groups, and analysis of variance (ANOVA) was used for comparison between the means of multiple groups. A P-value ≤0.05 was considered statistically significant.

### RNA Extraction and RNA-Seq Library Construction

This experiment was divided into in two groups including Aconitine group and Aconitine/AS-IV group, in order to obtain the DEGs of AS-IV on aconitine-induced cardiac damage. The effect of aconitine on gene expression was eliminated accordingly.

Total RNA extraction was performed with TRIZOL reagents from Invitrogen following the manufacturer's instructions. RNA library construction was then performed by BGI Co., Ltd, Shenzhen, China (http://www.genomics.cn/). An Agilent 2100 Bioanalyzer was used to detect RNA concentration, RNA Integrity Number (RIN) value, 28S/18S, and fragment size to determine the integrity of RNA, and the purity of RNA as detected using an ultraviolet spectrophotometer NanoDrop (OD260/280) (Qian et al., 2014).

### Bioinformatic Analysis of RNA-Seq Data

First, raw data from Illumina HiSeq sequencing were filtered, and clean reads were matched to the reference sequence. Based on the comparison results, differential splicing gene detection, single nucleotide polymorphisms (SNP), Indel detection, fusion gene detection, and other analyses were performed. Quantitative analysis was conducted on the known genes, and differential expression analysis was conducted according to the expression number of genes in different sample groups.

Second, for experiments without biological replication, we used the PossionDis algorithm to perform differential gene detection, and screened |log2(FoldChange)|>1&qvalue<0.001 as the DEGs. According to the result of DEGs, the heatmaps function (https://CRAN.R-project.org/package=pheatmap) in R software was used to performed clustering analysis.

Third, the DEGs were analyzed by a series of bioinformatics analyses such as Gene Ontology (GO) (Qian et al., 2014) function analysis (http://www.geneontology.org/), Kyoto Encyclopedia of Genes and Genomes (KEGG) (Kanehisa et al., 2019) signal pathway analysis (https://www.kegg.jp/), protein-protein interaction (PPI) (Szklarczyk et al., 2019) network prediction (http://string-db.org), and DNA-microRNA (Sticht et al., 2018) network analysis (http://mirwalk.umm.uni-heidelberg.de/). Finally, the obtained gene target network was imported into Cytoscape software (https://cytoscape.org/) for visual editing. CytoHubba (Chin et al., 2014) was employed to investigate node composition and pick out hub nodes with high degree of connectivity in the network.

### Validation of Hub Gene Targets

The expression of hub genes was verified in different transcriptomes. We retrieved the RNA-seq and miRNA-seq datasets from the GEO database (https://www.ncbi.nlm.nih.gov/geo/). Then RNA-seq series matrix files were submitted to Biojupies (http://biojupies.cloud), which was a web-automated generation application in the cloud. The samples in the dataset were divided into control group and the treatment group. All procedures followed the methods described in a previous paper (Torre et al., 2018). The limma package (Ritchie et al., 2015) in R software (http://www.bioconductor.org/packages/) was used for differential expression analysis of miRNA-seq microarray data.

The Sybyl X-2.0 (Tripos, St. Louis, MO, USA) (Jain, 2003) was employed to validate the compound-target association in our study. The compound was retrieved from ZINC database (http://zinc15.docking.org). The 3D (three dimensional) structures of target proteins were obtained from the RCSB-PDB database (http://www.rcsb.org/) and Uniprot (https://www.uniprot.org/) database. The proteins and ligands were preprocessed by the docking suite tool to remove water molecules, protonate 3D hydrogenation, fix termini treatment, and extract ligand substructure (Ragunathan et al., 2018). The docking mode was automatic and standard.

## Results

### Model of Cardiac Damage Induced by Aconitine

**Figure 2:**
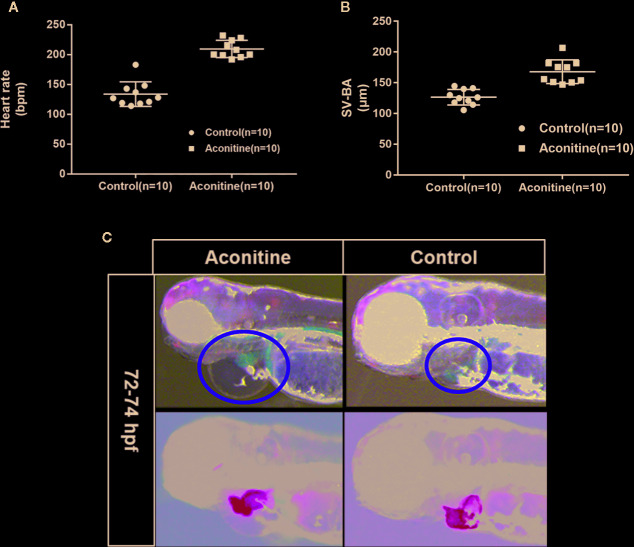
**(A)** Heart rates of zebrafish in aconitine group at 72 hpf (n = 10). **(B)** SV-BA distance, comparing control group with aconitine group (n = 10). **(C)** Effect of aconitine on the morphology of zebrafish heart.

A previous study has shown that at 72 hpf, the half maximal effective concentration (EC50) of the cardiac toxicity of aconitine to zebrafish embryos is 14.49 mg/L (Fang et al., 2012). The cardiac toxicity of aconitine is dose- and time-dependent. Our results show that the heart rates of the aconitine 15 mg/L group increased significantly compared with the control group (**Table 1**, **Figure 2A**) (P* < 0.01). Over time, some zebrafish manifested ventricular arrest or an irregular heartbeat. We also found that aconitine 15 mg/L caused statistically significant prolongation of the SV-BA distance (**Table 1**, **Figure 2B**) (P**<0.01). **Figure 2C** reveals that aconitine 15 mg/L caused the ventricle of the zebrafish to shrink, reduced pericardial edema, and led to accumulation of blood cells in the sinus veins and the dorsal aorta (yellow line), suggesting that aconitine can result in damage to the heart.

**Table 1:** Heart rates and SV-BA distance of zebrafish in the control and aconitine group at 72 hpf (n = 10).

Groups	Number	Heart rate (bpm) $\left(\bar{x}\pm s\right)$	SV-BA (μm) $\left(\bar{x}\pm s\right)$	P*	P**
**Aconitine**	10	207.2 ± 5.619*	207.2 ± 5.619**	<0.0001	<0.0001
**Control**	10	124.3 ± 2.119*	126.5 ± 3.988**		

*Compared with the control group, heart rates of the aconitine 15 mg/L group increased significantly. **Compared with the control group, the SB-BA distance of the aconitine 15 mg/L group increased significantly.

### Intervention of AS-IV

This experiment was divided into two parts. The first part was the screening of the safe dose range of AS-IV. The second part was the measurement of change in heart rate, SV-BA distance, and cardiac morphological features of zebrafish after AS-IV intervention.

**Figure 3:**
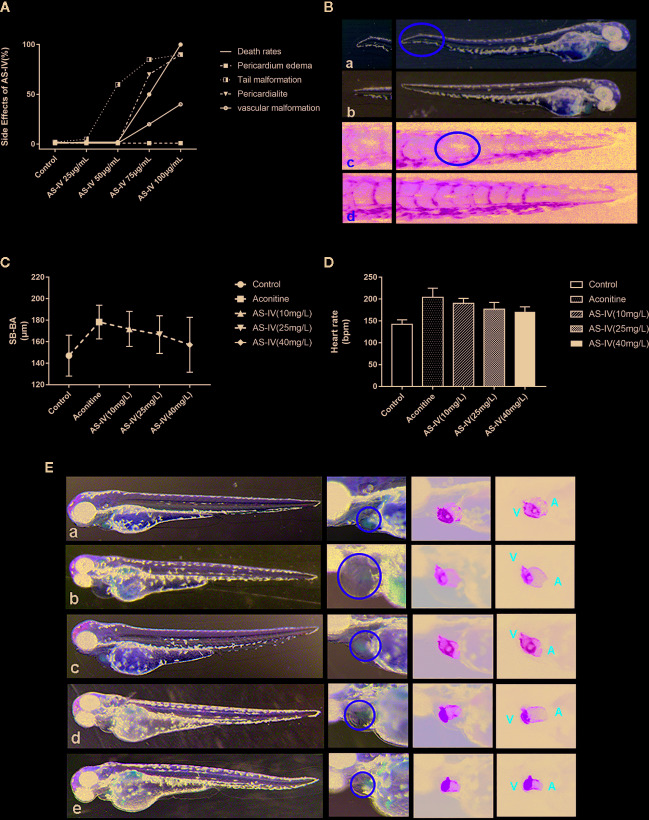
**(A)** Effects of different doses of AS-IV on mortality, and development of the heart and other parts of the zebrafish. **(B)** At 50 mg/L AS-IV, abnormal tail vascular development and tail malformation starts to become apparent. **(C)** Comparison of heart rates of zebrafish in each group (histogram). **(D)** Comparison of distance SV-BA in each group (trend graph). **(E)** Changes in heart morphology of zebrafish in each group. (a) Control group, (b) Aconitine group, (c) AS-IV 10 mg/L group, (d) AS-IV 25 mg/L group, (e) AS-IV 40 mg/L group.

In the first part, the testing concentrations of AS-IV were 25, 50, 75, and 100 mg/L (**Figure 3A**). A dose of 50 mg/L AS-IV saw the beginning of abnormal tail development of the zebrafish (**Figure 3B**), with no significant change in pericardial structure. Therefore, we selected 10, 25, and 40 mg/L as a reasonable concentration gradient for the later interventions.

In terms of the second part of the experiment, there were no statistically significant differences in heart rate between the AS-IV 10 mg/L group and the aconitine group (P* > 0.05). There was, however, a statistically significant difference between the AS-IV 25 and 40 mg/L groups compared with the aconitine group (P** < 0.01, P*** < 0.01) (**Table 2**, **Figure 3C**). In addition, there was no statistically significant difference in SV-BA distance between the AS-IV 10 mg/L and AS-IV 25 mg/L groups compared with the aconitine group (P* > 0.05, P** > 0.05). In contrast, there was a statistically difference between the AS-IV 40 mg/L group compared with the aconitine group (P*** < 0.05) (**Table 2**, **Figure 3D**).

**Table 2:** Heart rates and SV-BA distance of zebrafish in each group (n = 10).

Groups	Number	Heart rates (bpm) $\left(\bar{x}\pm s\right)$	SV-BA (μm) $\left(\bar{x}\pm s\right)$
**Control**	10	152.4 ± 2.0	147.1 ± 6.1
**Aconitine**	10	204.4 ± 6.6	178.5 ± 4.9
AS-IV 10 mg/L^*^	10	190.0 ± 3.6	171.8 ± 5.1
AS-IV 25 mg/L^**^	10	176.8 ± 4.9	166.6 ± 5.5
AS-IV 40 mg/L^***^	10	172.5 ± 3.2	157.4 ± 8.2
***F***	–	25.100	4.159
***P***	–	0.000	0.006

Compared with aconitine group, the heart rate of AS-IV 10mg/L is no statistically significant differences (P*>0.05), however, the heart rate of AS-IV 25mg/L and 40mg/L groups is significantly decreased. (P**<0.01, P***<0.01).Compared with aconitine group, the SV-BA of AS-IV 10mg/L and AS-IV 25mg/L groups is no statistically significant differences (P*>0.05, P**>0.05), however, the SV-BA of AS-IV 40mg/L group is decreased. (P***<0.05).

At 72 hpf, we can see that the pericardium of the zebrafish in the aconitine 15 mg/L group is clearly enlarged. **Figure 3E** shows that the pericardium boundary is close to the yolk sac. However, the extent of pericardial edema is reduced in the AS-IV groups (yellow line). Therefore, we can conclude that the cardioprotective effective AS-IV is dose-dependent, and in this experiment 40 mg/L AS-IV is the appropriate concentration for the intervention of aconitine-induced cardiac damage in zebrafish.

### Mapping of RNA-Seq Reads to Zebrafish Genome

In this section, the Illumina HiSeq platform was used to obtain the transcriptome sequence data of the AS-IV intervention in an aconitine-induced cardiac-damaged model of zebrafish. Clean reads (**Table 3**) obtained through filtration were statistically analyzed, including data volume statistics, base content distribution, and base mass distribution statistics. The utilization rate of the transcriptome data was directly reflected by the percentage of total mapped reads (**Table 4**) in total reads (>70%). We conclude, therefore, that the error rate in sequencing bases was low, and that the sequencing data are credible.

**Table 3:** Statistics for clean reads.

Clean reads	Clean reads^1^ (M)	Clean bases^2^ (G)	Q20 (%)	Q30 (%)	GC (%)	Read length (bp)
Aconitine_ AS-IV	40.0979M	6.0147G	98.37	95.63	48.55	150
Aconitine	40.1863M	6.0279G	98.35	95.57	48.67	150

^1^Clean reads (M); the number of clean reads obtained after filtration and in units of M.

^2^Clean bases (G): the number of clean reads obtained after filtration and in units of G.

**Table 4:** Statistics for mapped reads.

Mapped reads	Total reads^1^	Total mapped^2^	Reads map to+	Reads map to−	Splice mapped
Aconitine_ AS-IV	40097856	32334002 (80.64%)	16182534 (40.36%)	16151468 (40.28%)	17374239 (43.33%)
Aconitine	40186304	32830290 (81.70%)	16409274 (40.83%)	16421016 (40.86%)	17253601 (42.93%)

1 Total reads: the number and percentage of reads mapped to the genome.

2 Total mapped: the percentage of total mapped reads in total reads should be more than 70%.

### Differential Expression Analysis

**Figure 4:**
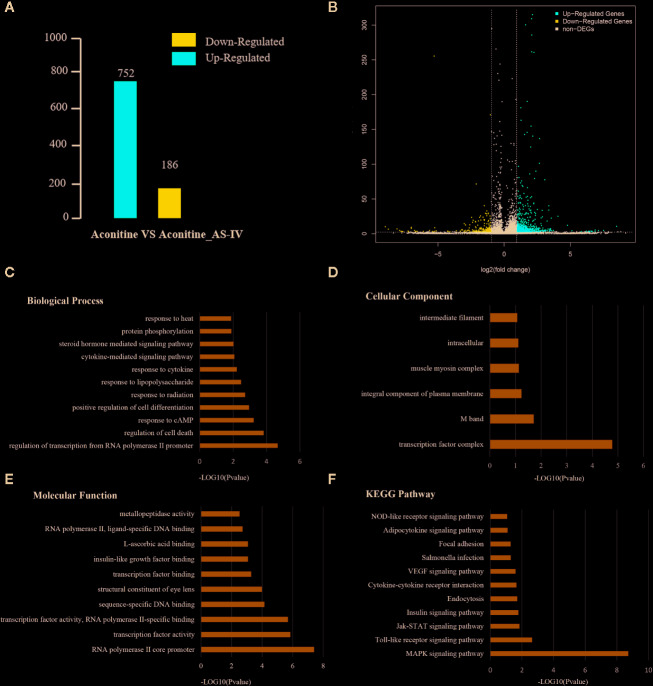
**(A)** Statistical chart of the number of DEGs. The horizontal axis represents comparison of different samples, the vertical axis represents the number of DEGs, red represents up-regulation, blue represents down-regulation. **(B)** Volcano Plot of DEGs. Each dot in the volcano plot of DEGs represents a gene. The red dots in the plot represent up-regulated DEGs, the blue dots represent down-regulated DEGs, and the black dots represent non-differentially expressed genes. The dotted line means the threshold lines of FDR and fold change **(C)** Biological Process diagram of DEGs. X axis represents -log10 (p-value). Y axis represents enrichment names. **(D)** Cellular component diagram of DEGs. **(E)** Molecular function diagram of DEGs. **(F)** Diagram of KEGG pathway.

Under different experimental conditions, genes with significant differences in expression level are called DEGs. The results of DEGs showed that a total of 938 genes were obtained **(Supplementary Material, Table 1)** of which 752 were up-regulated and 186 were down-regulated (**Figure 4A**). The differences of gene expression levels and the statistical significance in the two comparison samples are rapidly examined by the Volcano Plot. The differential expression between aconitine/AS-IV group and aconitine group in is shown as a volcano plot in the **Figure 4B**.

### Gene Ontology and KEGG Pathway Enrichment Analysis of DEGs

Gene ontology is the international standard classification system of gene function. Through the GO enrichment analysis of the differentially enriched genes, their GO classifications and gene functions can be found. The GO annotation system contains three main branches: namely, a biological process, a molecular function, and a cellular component (**Figures 4C–E**). The GO enrichment analysis of the DEGs was performed, and the results of top five items in three main branches includes: biological process contains regulation of transcription from RNA polymerase II promoter, regulation of cell death, response to cAMP, positive regulation of cell differentiation, response to radiation. The items that relate to molecular function are RNA polymerase II core promoter proximal region sequence-specific DNA binding, sequence-specific DNA binding, RNA polymerase II core promoter proximal region sequence-specific binding, sequence-specific DNA binding, structural constituent of eye lens. The items that relates to cellular component are transcription factor complex M band, integral component of plasma membrane, muscle myosin complex, intracellular.

Similar to the GO enrichment principle, KEGG pathway significance enrichment can determine the most important biochemical metabolic pathways and signal transduction pathways involved in DEGs (**Figure 4F**). A scatter diagram is a graphical presentation of the results of the KEGG enrichment analysis. The top five of these are mitogen activated protein kinase (MAPK) signaling pathway, toll-like receptor signaling pathway, Janus kinase-signal transducers and activators of transcription (JAK-STAT) signaling pathway, insulin signaling pathway, and endocytosis. The enrichment degree of MAPK signaling pathway is much higher other signaling pathways, so it is considered as a key signaling pathway in cardioprotective effect of AS-IV.

### Network Analysis From Protein-Protein Interaction Data

**Figure 5:**
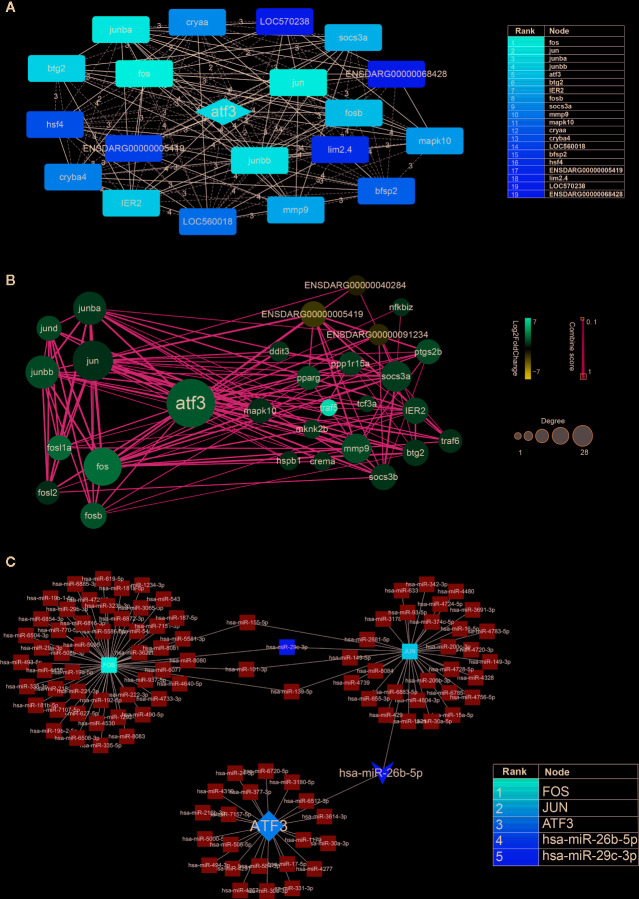
**(A)** Hub genes identified by Cytoscape network analysis. The shade of the color indicates the degree of the gene. The list on the right is the result of the top 20 genes. **(B)** Protein-protein interaction data network of ATF3. Red, up-regulation; blue, down-regulation. Color depth and size of the dots were proportional to the fold change and correlated degree, respectively; thickness of the line between the two nodes indicated the connection strength positively. **(C)** DNA-microRNA network of ATF3. The shade of the color indicates the degree of the genes. The list on the right is the result of the top 5 genes.

First, we inputted DEGs into a STRING database to obtain a protein-protein interaction (PPI) network **(Supplementary Material, Data Sheet 1)**. The network was then imported into Cytoscape software, and core genes were extracted using the Cytohubba. The top 20 genes were screened out, of which the top five were *FOS*, *JUN*, *JUNBA*, *JUNBB*, and *ATF3*. From these genes, we can see that *JUN*, *JUNBA*, and *JUNBB* are all immune response complexes, and that *ATF3* is of particular significance as an upstream regulator (**Figure 5A**). The log2(FoldChange) value (Aconitine *vs* Aconitine/AS-IV) of *ATF3* and *JUN* is 2.168 and 1.122, respectively.

Second, we took *ATF3* as the center, screened out the genes interacting with it, and then constructed an *ATF3* gene regulatory network, which demonstrated the upstream and downstream regulatory relationship of *ATF3* more intuitively. These genes included immune response complexes (*FOS*, *JUN*, *MMP9*), cytokine signal inhibitors (*SOCS3A*, *SOCS3B*), and genes involved in regulating the cell cycle (*MAPK10*, *TRAF5*, *TRAF6*) (**Figure 5B**).

It is known that miRNA plays an important role in regulating cardiac homeostasis; therefore, to further study the miRNA related to *ATF3*, we constructed a DNA-microRNA network based on *FOS*, *JUN*, *JUNBA*, *JUNBB*, and *ATF3*, and screened the top miRNA using the Cytohubba plug-in. The results show that the miRNA with the highest core gene degree was *miR-26b-5p*, and that *JUN* could also be regulated. Therefore, we believe that the hub genes might be *miR-26b-5p/ATF3/JUN* (**Figure 5C**).

### Validation of Hub Targets *miR-26b-5p/ATF3/JUN*

The end stage of many CVDs such as heart failure, myocardial infarction, the heart is prone to myocardial fibrosis, cardiac enlargement, and other cardiac damage symptoms (Tzahor and Poss, 2017; Yuan and Braun, 2017). There is currently no known effective prophylaxis for myocardial damage. The present study demonstrates that *miR-26b-5p/ATF3/JUN* may be the potential targets of cardiac protection through RNA-seq and a series of bioinformatics analyses.

First, RNA-seq dataset (GSE108157) of left ventricle samples in 11 patients with end-stage heart failure was analyzed by Biojupies to obtain the DEGs. We found by differential expression analysis that there were 298 overlapping DEGs in zebrafish and human samples. Interestingly, the top five genes (*FOS*, *JUN*, *JUNBA*, *JUNBB*, and *ATF3*) screened in the zebrafish were all included in these DEGs. Furthermore, *ATF3* and *JUN* are up-regulated in zebrafish, while in patients they are down-regulated (**Table 5**). Furthermore, the RNA-seq matrix of myocardial infarction tissue in 20 mice was screened from the GEO database (GSE23294), then the differential expression analysis was performed to further verify the expression of *ATF3* and *JUN*. The results suggested that *ATF3* and *JUN* were both downregulated in heart damage tissue of mouse and human (**Table 5**). Finally, miRNA-seq transcriptomic dataset with 48 heart failure patients and 32 healthy donors (GSE136547) was analyzed to evaluate the expression of *miR-26b-5p.* Compared with healthy persons, *miR-26b-5p* was upregulated in the heart failure patients (**Table 5**). The results of this experiment partly confirm that *miR-26b-5p/ATF3/JUN* may play a considerable role in cardiac protection.

**Table 5:** Hub genes expression in different transcriptome sequence datasets.

Genes	Sample type	Log2FC	Regulation
atf3	Zebrafish	2.168584	Up
jun	Zebrafish	1.122770	Up
ATF3	Mouse	−0.092700	Down
JUN	Mouse	−0.234537	Down
ATF3	Human	−0.583325	Down
JUN	Human	−0.350524	Down
miRNA-26b-5p	Human	0.118979	Up

With the aim to improving the accuracy of the results, virtual screening was further performed to assess the binding affinity between AS-IV and *ATF3/JUN*. SYBYL X 2.0 molecular docking experiment was exploited to elaborate the potential relationship between protein and compound based on the Cscore. When Cscore value <3, it means that the binding affinity is weak. The formation of the hydrogen bond was of vital importance for any stable interaction between compounds and proteins. The information of Cscore and hydrogen bond were shown in **Table 6**. It is concluded that AS-IV has a stable interaction with *ATF3/JUN*.

**Table 6:** Molecular docking results of AS-IV and ATF3/JUN.

Compound	Structure	Cscore (Value)		Hydrogen bond (Number)	
		JUN	ATF3	JUN	ATF3
AS-IV	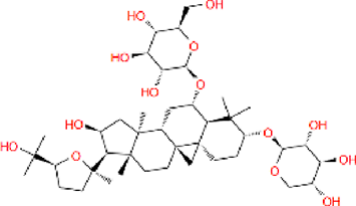	4	5	2	3

## Discussion

Our study demonstrated that AS-IV can improve the manifestations of aconitine-induced cardiac damage, such as increased heart rate, pericardial edema, and increased SV-BA interval induced by aconitine in zebrafish. Other questions addressed by this study include: Do different concentrations of AS-IV affect the hearts of zebrafish? What is the safe concentration range of AS-IV? Further work indicated at an AS-IV concentration of 50 mg/L, abnormal tail development in the zebrafish was first observed, with the abnormality most commonly being a warped tail. No pericardial edema or other cardiac damage was observed in zebrafish at different concentrations of AS-IV. Within the safe drug concentration range, we finally determined that the appropriate concentration of AS-IV for aconitine-induced cardiac damage is 40 mg/L. Through statistical analysis, we find that AS-IV at different concentrations could reduce the increase in heart rate induced by aconitine, and reduce the degree of pericardial edema and SV-BA spacing in a dose-dependent manner. Therefore, our data suggest that AS-IV has a protective effect on cardiac damage induced by aconitine in zebrafish.

The DEGs of AS-IV intervention in aconitine-induced cardiac damage were identified. Of a total of 938 genes, 752 were up-regulated and 186 were down-regulated, according to high-throughput sequencing technology. To further understand these DEGs, a PPI network of DEGs was constructed to screen out the top 20 core genes, among which the top five genes were *FOS*, *JUN*, *JUNBA*, *JUNBB*, and *ATF3*. Among these five genes, *FOS*, *JUN*, *JUNBA*, and *JUNBB* were immune response factors, while *ATF3*, as the upstream regulatory gene, was of particular significance. In order to further study the genes with a specific interaction with *ATF3*, 29 important proteins from the PPI network were predicted which were closely related to *ATF3*, including proteins involved in the immune response (*JUN*, *JUN*B, *JUND*, *FOS*, etc.) Given that miRNA is an important regulatory molecule in the gene regulatory network, we further constructed a DNA-microRNA interaction network, among which *miR-26b-5p* had the highest binding degree. Therefore, *miR-26b-5p*/*ATF3*/*JUN* became the hub genes in this study.

To obtain reliable evidence regarding the relationship between the expression of *miR-26b-5p*/*ATF3*/*JUN* and cardiac damage-related diseases, we further verify the expression of *miR-26b-5p*/*ATF3*/*JUN* in human and mouse cardiac damage tissue in the GEO database. By analyzing and comparing the DEGs, we find that the expression of *miR-26b-5p*/*ATF3*/*JUN* in cardiac damage specimens is just opposite to the regulation of AS-IV cardioprotective effect in this study. Furthermore, molecular docking experiment was performed to elaborate the potential relationship and the hydrogen bond formation between AS-IV and *ATF3*/*JUN*. Therefore, it can be considered that AS-IV may play a role in cardiac protection by regulating *miR-26b-5p*/*ATF3*/*JUN*.

Although it is well recognized that *ATF3* protein represents one of the 53 basic leucine zipper (b-Zip) transcription factors in humans, and that the transcriptional activation or repression activity of *ATF3* is located at both the N- or C-terminal region, the detailed mechanism of this activity remains elusive (Hai and Hartman, 2001; Hartman et al., 2004). As the “hub” of the cell adaptive response, *ATF3* can activate or inhibit target gene transcription when it binds with different structures to form homologous or heterologous dimers (Hai et al., 2010; Zhou et al., 2014). This may be why *ATF3* plays a dual role in heart disease. Recent studies demonstrate that activation of *ATF3* expression improves CVD. Up-regulation of *ATF3* in cardiac fibroblasts is a compensatory mechanism for self-protection during hypertensive ventricular remodeling and heart failure. To further explore the mechanism of the cardioprotective effect of *ATF3*, research shows that *ATF3* inhibits the target gene Map2K3 to mediate cardioprotection, and further inhibits the p38-TGF-β signaling pathway (Li et al., 2017b). Another study also reveals that *ATF3* expression in cardiomyocytes can improve cardiac function. This process is achieved by dampening the inflammatory responses and inhibiting the expression of extracellular matrix (ECM) remodeling genes, and can also control peripheral glucose tolerance to preserve homeostasis in the heart (Kalfon et al., 2017).

On the contrary, Zhou et al. (2011) first reported that *ATF3* deficiency promotes myocardial hypertrophy and fibrosis in heart failure caused by excessive stress load, suggesting that *ATF3* has a cardioprotective function. Research by Heng Lin indicates that *ATF3* therapy, or the use of the *ATF3*-inducer, tert-Butylhydroquinone, protects against pressure-overload heart failure by inhibiting Beclin-1-associated detrimental autophagic activity (Lin et al., 2014). In a word, many phenomena cannot simply be explained by up-regulation or down-regulation; they are more like the concept of Yin and Yang in Chinese philosophical theory, in which the ultimate goal is to pursue a balance between Yin and Yang. The result of the present study will provide a novel evidence for the cardioprotective effect of *ATF3* expression.

*JUN* is a family of protein kinases in the MAPK signal transduction cascade. It is activated by double phosphorylation of tyrosine and threonine residues, enters the nucleus, regulates the expression of specific genes, and participates in the regulation of cell apoptosis and other physiological activities (Kyriakis et al., 1994; Huang et al., 2004; Sun et al., 2019). Conversely, c−*JUN* has also been demonstrated to have anti−apoptotic properties. He et al. found that up-regulation of miR-138 can reduce hypoxia-induced apoptosis *via* up-regulating the *MLK3/JNK/c-Jun* signaling pathway in cardiac muscle samples of patients with congenital heart disease (He et al., 2013). Renata Windak et al. (Windak et al., 2013) investigated the function of *c-Jun*, and the result suggested that it can prevent stress-imposed maladaptive remodeling of the heart in mice. One possible explanation for these experiments is that the role of *c−Jun* in promoting survival and apoptosis may be the adaptation of myocardial tissue to chronic hypoxia; that is, some mechanism of self-protection and self-repair.

Collectively, *miRNA-26b* also plays an important role in the cardiovascular system. In terms of clinical studies, different expressions of three miRNAs (*miR-26b-5p*, *miR-660-5p*, and *miR-320a*) were observed in patients with myocardial infarction and healthy individuals (Jakob et al., 2017). In vitro study demonstrated that *miR-26b-5p* regulates cell proliferation to suppresses angiogenesis in hepatocellular carcinoma. *Vivo* studies have shown similar results. The latest research (Qi et al., 2020) demonstrated that downregulation of *miR-26b-5p* expression can facilitate exercise-induced physiological cardiac hypertrophy by augmenting autophagy in rats. Han et al. (2012) underscore the functional relevance of *miR-26b-5p* in regulating gene expression during cardiac hypertrophy.

## Conclusion

In summary, we have provided evidence that AS-IV shows antagonistic action against arrhythmia induced by aconitine in zebrafish, and could also improve structural damage in zebrafish hearts, such as reducing the SV-BA distance and relieving pericardial edema, in order to play a protective role in the heart. Mechanistically, *via* the RNA-seq and bioinformatics analysis of DEGs in zebrafish, it is found that *miR-26b-5p*/*ATF3*/*JUN* and MAPK signaling pathway were identified as hub gene targets and key signaling pathway, respectively. The present study represents new evidence and a novel therapeutic approach of cardioprotective effect of AS-IV on zebrafish. However, although zebrafish have certain benefits in studying heart disease, mammals or human studies are necessary to explore the pharmacological mechanisms of AS-IV and aconitine-induced cardiac damage at a deeper level. Systematic relationship between hub gene targets is required to discuss in further *vitro* and *vivo* experiments.

## Data Availability Statement

The datasets [GSE108157/ GSE23294/GSE136547] for this study can be found in the GEO database [https://www.ncbi.nlm.nih.gov/geo/].

## Author Contributions

All authors contributed to the article and approved the submitted version. MW and YS have contributed equally to this work.

## Funding

This study was supported by the National Natural Science Foundation of China (8177141253), key specialty cultivation project of TCM (2018-2020), and Shanghai University of Traditional Chinese Medicine (project code:2019LK022).

## Conflict of Interest

The authors declare that the research was conducted in the absence of any commercial or financial relationships that could be construed as a potential conflict of interest.

## Supplementary Material

Table 1 The DEGs of Aconitine VS Aconitine_Astragaloside.

Data Sheet 1 Protein-protein interaction network of DEGs from STRING database.

Video 1 The video of heartbeats of zebrafish.

## References

[B1] Asnani A. Peterson R. T. (2014). The zebrafish as a tool to identify novel therapies for human cardiovascular disease. Dis. Model Mech. 7 (7), 763–767. 10.1242/dmm.016170 24973746 PMC4073266

[B2] Benjamin E. J. Virani S. S. Callaway C. W. Chamberlain A. M. Chang A. R. Cheng S. (2018). Heart Disease and Stroke Statistics-2018 Update: A Report From the American Heart Association. Circulation 137 (12), e67–e492. 10.1161/cir.0000000000000558 29386200

[B3] Cheng S. Zhang X. Feng Q. Chen J. Shen L. Yu P. (2019). Astragaloside IV exerts angiogenesis and cardioprotection after myocardial infarction via regulating PTEN/PI3K/Akt signaling pathway. Life Sci. 227, 82–93. 10.1016/j.lfs.2019.04.040 31004658

[B4] Chin C. H. Chen S. H. Wu H. H. Ho C. W. Ko M. T. Lin C. Y. (2014). cytoHubba: identifying hub objects and sub-networks from complex interactome. BMC Syst. Biol. 8 Suppl 4 (Suppl 4), S11. 10.1186/1752-0509-8-s4-s11 25521941 PMC4290687

[B5] Conesa A. Madrigal P. Tarazona S. Gomez-Cabrero D. Cervera A. McPherson A. (2016). Erratum to: A survey of best practices for RNA-seq data analysis. Genome Biol. 17 (1), 181. 10.1186/s13059-016-1047-4 27565134 PMC5000515

[B6] Du Z. Wang G. Gao S. Wang Z. (2015). Aryl organophosphate flame retardants induced cardiotoxicity during zebrafish embryogenesis: by disturbing expression of the transcriptional regulators. Aquat. Toxicol. 161, 25–32. 10.1016/j.aquatox.2015.01.027 25661707

[B7] Fang F. Jie Z. Linzhong Y. Jiabo L. (2012). Priliminary investigation of cardiac toxicity to zebrafish embryo by Aconitine. Pharmacol. Clinics Chin. Mater. Med. 28 (2), 11. 10.13412/j.cnki.zyyl.2012.02.018

[B8] Gut P. Reischauer S. Stainier D. Y. R. Arnaout R. (2017). Little fish, big data: zebrafish as a model for cardiovascular and metabolic diease. Physiol. Rev. 97 (3), 889–938. 10.1152/physrev.00038.2016 28468832 PMC5817164

[B9] Hai T. Hartman M. G. (2001). The molecular biology and nomenclature of the activating transcription factor/cAMP responsive element binding family of transcription factors: activating transcription factor proteins and homeostasis. Gene 273 (1), 1–11. 10.1016/s0378-1119(01)00551-0 11483355

[B10] Hai T. Wolford C. C. Chang Y. S. (2010). ATF3, a hub of the cellular adaptive-response network, in the pathogenesis of diseases: is modulation of inflammation a unifying component? Gene Expr. 15 (1), 1–11. 10.3727/105221610x12819686555015 21061913 PMC6043823

[B11] Han M. Yang Z. Sayed D. He M. Gao S. Lin L. (2012). GATA4 expression is primarily regulated via a miR-26b-dependent post-transcriptional mechanism during cardiac hypertrophy. Cardiovasc. Res. 93 (4), 645–654. 10.1093/cvr/cvs001 22219180 PMC3291090

[B12] Hartman M. G. Lu D. Kim M. L. Kociba G. J. Shukri T. Buteau J. (2004). Role for activating transcription factor 3 in stress-induced beta-cell apoptosis. Mol. Cell Biol. 24 (13), 5721–5732. 10.1128/mcb.24.13.5721-5732.2004 15199129 PMC480886

[B13] He S. Liu P. Jian Z. Li J. Zhu Y. Feng Z. (2013). miR-138 protects cardiomyocytes from hypoxia-induced apoptosis via MLK3/JNK/c-jun pathway. Biochem. Biophys. Res. Commun. 441 (4), 763–769. 10.1016/j.bbrc.2013.10.151 24211202

[B14] Howe K. Clark M. D. Torroja C. F. Torrance J. Berthelot C. Muffato M. (2013). The zebrafish reference genome sequence and its relationship to the human genome. Nature 496 (7446), 498–503. 10.1038/nature12111 23594743 PMC3703927

[B15] Huang C. Jacobson K. Schaller M. D. (2004). MAP kinases and cell migration. J. Cell Sci. 117 (Pt 20), 4619–4628. 10.1242/jcs.01481 15371522

[B16] Jain A. N. (2003). Surflex: fully automatic flexible molecular docking using a molecular similarity-based search engine. J. Med. Chem. 46 (4), 499–511. 10.1021/jm020406h 12570372

[B17] Jakob P. Kacprowski T. Briand-Schumacher S. Heg D. Klingenberg R. Stähli B. E. (2017). Profiling and validation of circulating microRNAs for cardiovascular events in patients presenting with ST-segment elevation myocardial infarction. Eur. Heart J. 38 (7), 511–515. 10.1093/eurheartj/ehw563 28011706

[B18] Kalfon R. Koren L. Aviram S. Schwartz O. Hai T. Aronheim A. (2017). ATF3 expression in cardiomyocytes preserves homeostasis in the heart and controls peripheral glucose tolerance. Cardiovasc. Res. 113 (2), 134–146. 10.1093/cvr/cvw228 28082453

[B19] Kanehisa M. Sato Y. Furumichi M. Morishima K. Tanabe M. (2019). New approach for understanding genome variations in KEGG. Nucleic Acids Res. 47 (D1), D590–d595. 10.1093/nar/gky962 30321428 PMC6324070

[B20] Keßler M. Berger I. M. Just S. Rottbauer W. (2015). Loss of dihydrolipoyl succinyltransferase (DLST) leads to reduced resting heart rate in the zebrafish. Basic Res. Cardiol. 110 (2), 14. 10.1007/s00395-015-0468-7 25697682 PMC4335124

[B21] Kyriakis J. M. Banerjee P. Nikolakaki E. Dai T. Rubie E. A. Ahmad M. F. (1994). The stress-activated protein kinase subfamily of c-Jun kinases. Nature 369 (6476), 156–160. 10.1038/369156a0 8177321

[B22] Li L. C. Xu L. Hu Y. Cui W. J. Cui W. H. Zhou W. C. (2017a). Astragaloside IV Improves Bleomycin-Induced Pulmonary Fibrosis in Rats by Attenuating Extracellular Matrix Deposition. Front. Pharmacol. 8, 513. 10.3389/fphar.2017.00513 28848434 PMC5550738

[B23] Li Y. Li Z. Zhang C. Li P. Wu Y. Wang C. (2017b). Cardiac Fibroblast-Specific Activating Transcription Factor 3 Protects Against Heart Failure by Suppressing MAP2K3-p38 Signaling. Circulation 135 (21), 2041–2057. 10.1161/circulationaha.116.024599 28249877 PMC5542579

[B24] Li G. M. Zhang C. L. Rui R. P. Sun B. Guo W. (2018). Bioinformatics analysis of common differential genes of coronary artery disease and ischemic cardiomyopathy. Eur. Rev. Med. Pharmacol. Sci. 22 (11), 3553–3569. 10.26355/eurrev_201806_15182 29917210

[B25] Lin H. Li H. F. Chen H. H. Lai P. F. Juan S. H. Chen J. J. (2014). Activating transcription factor 3 protects against pressure-overload heart failure via the autophagy molecule Beclin-1 pathway. Mol. Pharmacol. 85 (5), 682–691. 10.1124/mol.113.090092 24550138

[B26] Lin J. Fang L. Li H. Li Z. Lyu L. Wang H. (2019). Astragaloside IV alleviates doxorubicin induced cardiomyopathy by inhibiting NADPH oxidase derived oxidative stress. Eur. J. Pharmacol. 859, 172490. 10.1016/j.ejphar.2019.172490 31229536

[B27] Qi J. Luo X. Ma Z. Zhang B. Li S. Zhang J. (2020). Downregulation of miR-26b-5p, miR-204-5p, and miR-497-3p Expression Facilitates Exercise-Induced Physiological Cardiac Hypertrophy by Augmenting Autophagy in Rats. Front. Genet. 11, 78. 10.3389/fgene.2020.00078 32140172 PMC7042403

[B28] Qian X. Ba Y. Zhuang Q. Zhong G. (2014). RNA-Seq technology and its application in fish transcriptomics. Omics 18 (2), 98–110. 10.1089/omi.2013.0110 24380445 PMC3920896

[B29] Ragunathan A. Malathi K. Ramaiah S. Anbarasu A. (2018). FtsA as a cidal target for Staphylococcus aureus: Molecular docking and dynamics studies. J. Cell Biochem. 120 (5), 7751–7758. 10.1002/jcb.28049 30417432

[B30] Ren S. Zhang H. Mu Y. Sun M. Liu P. (2013). Pharmacological effects of Astragaloside IV: a literature review. J. Tradit. Chin. Med. 33 (3), 413–416. 10.1016/s0254-6272(13)60189-2 24024343

[B31] Ritchie M. E. Phipson B. Wu D. Hu Y. Law C. W. Shi W. (2015). limma powers differential expression analyses for RNA-sequencing and microarray studies. Nucleic Acids Res. 43 (7), e47. 10.1093/nar/gkv007 25605792 PMC4402510

[B32] Sticht C. De La Torre C. Parveen A. Gretz N. (2018). miRWalk: An online resource for prediction of microRNA binding sites. PloS One 13 (10), e0206239. 10.1371/journal.pone.0206239 30335862 PMC6193719

[B33] Sui Y. B. Wang Y. Liu L. Liu F. Zhang Y. Q. (2019). Astragaloside IV alleviates heart failure by promoting angiogenesis through the JAK-STAT3 pathway. Pharm. Biol. 57 (1), 48–54. 10.1080/13880209.2019.1569697 30905241 PMC8871603

[B34] Sun Y. Zhang D. Guo X. Li W. Li C. Luo J. (2019). MKK3 modulates JNK-dependent cell migration and invasion. Cell Death Dis. 10 (3), 149. 10.1038/s41419-019-1350-6 30770795 PMC6377636

[B35] Szklarczyk D. Gable A. L. Lyon D. Junge A. Wyder S. Huerta-Cepas J. (2019). STRING v11: protein-protein association networks with increased coverage, supporting functional discovery in genome-wide experimental datasets. Nucleic Acids Res. 47 (D1), D607–d613. 10.1093/nar/gky1131 30476243 PMC6323986

[B36] Torre D. Lachmann A. Ma'ayan A. (2018). BioJupies: Automated Generation of Interactive Notebooks for RNA-Seq Data Analysis in the Cloud. Cell Syst. 7 (5), 556–561.e553. 10.1016/j.cels.2018.10.007 30447998 PMC6265050

[B37] Tzahor E. Poss K. D. (2017). Cardiac regeneration strategies: Staying young at heart. Science 356 (6342), 1035–1039. 10.1126/science.aam5894 28596337 PMC5614484

[B38] Westerfield M. (1994). The zebrafish book: a guide for the laboratory use of zebrafish Danio (Brachydanio) rerio (Eugene: University of Oregon Press).

[B39] Wilson J. M. Bunte R. M. Carty A. J. (2009). Evaluation of rapid cooling and tricaine methanesulfonate (MS222) as methods of euthanasia in zebrafish (Danio rerio). J. Am. Assoc. Lab. Anim. Sci. 48 (6), 785–789. 19930828 PMC2786934

[B40] Windak R. Müller J. Felley A. Akhmedov A. Wagner E. F. Pedrazzini T. (2013). The AP-1 transcription factor c-Jun prevents stress-imposed maladaptive remodeling of the heart. PloS One 8 (9), e73294. 10.1371/journal.pone.0073294 24039904 PMC3769267

[B41] Yuan X. Braun T. (2017). Multimodal Regulation of Cardiac Myocyte Proliferation. Circ. Res. 121 (3), 293–309. 10.1161/circresaha.117.308428 28729454

[B42] Zang Y. Wan J. Zhang Z. Huang S. Liu X. Zhang W. (2020). An updated role of astragaloside IV in heart failure. BioMed. Pharmacother. 126, 110012. 10.1016/j.biopha.2020.110012 32213428

[B43] Zhao J. Yang P. Li F. Tao L. Ding H. Rui Y. (2012). Therapeutic effects of astragaloside IV on myocardial injuries: multi-target identification and network analysis. PloS One 7 (9), e44938. 10.1371/journal.pone.0044938 23028693 PMC3444501

[B44] Zhou H. Shen D. F. Bian Z. Y. Zong J. Deng W. Zhang Y. (2011). Activating transcription factor 3 deficiency promotes cardiac hypertrophy, dysfunction, and fibrosis induced by pressure overload. PloS One 6 (10), e26744. 10.1371/journal.pone.0026744 22053207 PMC3203896

[B45] Zhou H. Guo H. Zong J. Dai J. Yuan Y. Bian Z. Y. (2014). ATF3 regulates multiple targets and may play a dual role in cardiac hypertrophy and injury. Int. J. Cardiol. 174 (3), 838–839. 10.1016/j.ijcard.2014.04.160 24794959

