# Supplementary material for: Identification of Hub Genes in Protective Effect of Astragaloside IV on Aconitine-Induced Cardiac Damage in Zebrafish Based on Bioinformatics Analysis
Source: Front Pharmacol. 2020 Jun 24;11:957. doi: 10.3389/fphar.2020.00957 (PMC7327619; doi:10.3389/fphar.2020.00957)
Supplement: Supplementary file 4 [file DataSheet_2.docx]

SUPPLEMENTARY MATERIAL

1. DEGs of Aconitine-VS-Aconitine_Astragaloside
2. The STRING interactions of DEGs
3. Raw data of patients with cardiac damage
4. The DEGs of validation in zebrafish and patients
5. The video of heartbeats of zebrafish
